# Editorial: Advances in epilepsy research: exploring biomarkers, brain stimulation, and neurosurgical interventions

**DOI:** 10.3389/fnhum.2025.1632002

**Published:** 2025-06-12

**Authors:** Kazuki Sakakura, Joel Oster

**Affiliations:** ^1^Department of Neurosurgery, Rush University Medical Center, Chicago, IL, United States; ^2^Department of Neurology, Tufts University School of Medicine and Tufts Medical Center, Boston, MA, United States

**Keywords:** epilepsy, biomarkers, neurostimulation, neuromodulation, neurosurgical interventions

## Introduction

Epilepsy remains one of the most prevalent and complex neurological disorders, affecting 1–2% people worldwide (Falco-Walter, 2020). Despite new medications, significant subsets of patients (~35 %) remain medically refractory, necessitating innovative diagnostic and therapeutic approaches (Ben-Menachem, 2014; Janson and Bainbridge, 2021). This Research Topic on Neuromodulation aims to highlight recent developments in biomarkers, neuroimaging, brain stimulation, and surgical interventions, providing insights into both mechanistic understanding and clinical applications.

Neuromodulation is an evolving field with FDA approval in the US for treating epilepsy with Vagal Nerve Stimulation, Responsive cortical stimulation, and Deep Brain Stimulation of the anterior thalamic nucleus although in Europe, Responsive cortical stimulation is not approved for treating epilepsy although the other applications are approved (Morrell and Denison, 2022). Focal epilepsies are being investigated for treatments with TMS and tDCS, and idiopathic generalized onset seizures and related syndromes such as in Lennox-Gastaut syndrome are being investigated with DBS in the Anterior nucleus, the centromedianum nucleus, and the pulvinar nuclei of the thalamus (Morrell and Denison, 2022; Nanda et al., 2024; Kalamatianos et al., 2023).

## Electrophysiological and neuroimaging biomarkers advances in epilepsy

Recent developments in biomarkers and neuroimaging provide a deeper understanding of ictogenesis, seizure propagation, and surgical target identification. Electrophysiological biomarkers of epilepsy using electroencephalography (EEG) have been explored since the discovery by Hans Berger in the1930s, with scalp EEG serving as a primary tool for seizure detection and analysis (Crone et al., 1998). Interictal and ictal discharges were observed subsequent to EEG's discovery and implantation. By the 1990s, studies noted that high-gamma oscillations observed through intracranial electrodes were associated with epileptogenicity (Chen et al., 2021; Zweiphenning et al., 2022). Since the 2010s, phase-amplitude coupling, in which high-gamma oscillations are nested within slow waves, has been also recognized as a key biomarker of epilepsy (Motoi et al., 2018; Sakakura et al., 2023). Among the critical electrophysiological biomarkers of epilepsy, interictal spikes are particularly noteworthy (Asano et al., 2009). Previous studies have suggested the localizing value of interictal spikes and are complementary with in evaluating high-gamma oscillations (Zweiphenning et al., 2022). Within this context, Lu, Yang et al. have recently highlighted an important finding using stereotactic-EEG (SEEG) in patients with mesial temporal lobe epilepsy. Their study focuses on morphological changes in preictal and interictal spike patterns, demonstrating that the emergence of hypersynchronous transients in clusters serves as a crucial indicator of transition from the interictal to the ictal state.

In recent years, functional MRI (fMRI) has been increasingly utilized as a neuroimaging biomarker for epilepsy since fMRI detects functional brain regions by measuring blood-oxygen-level-dependent signals and might be incrementally useful in the presurgical evaluation for epilepsy (Binder et al., 2008). Beyond this application, recent advancements have leveraged resting-state fMRI to analyze large-scale brain networks, allowing for a more comprehensive assessment of functional connectivity (Bettus et al., 2010). One of the key advantages of fMRI is its non-invasive nature, which allows for example study of comparisons between drug-resistant epilepsy patients and either healthy controls or drug-responsive epilepsy patients. This approach has facilitated investigations into the relationship between altered functional connectivity and cognitive impairment in drug-resistant epilepsy patients (Ibrahim et al., 2014; Jiang et al., 2018). Furthermore, some studies have suggested that functional connectivity assessments may serve as biomarkers for treatment resistance (Kay et al., 2013). In this topic, Li et al. have demonstrated changes in functional connectivity strength between the temporal lobe and the prefrontal cortex in drug-resistant epilepsy patients. Their findings suggest that the left caudate nucleus may serve as a potential target for improving both cognitive dysfunction and seizure control.

## Advancements in brain stimulation and neurosurgical interventions

Prior to surgical treatment for drug-resistant epilepsy, intracranial electrode placement might be performed to identify both the seizure onset zone and critical functional areas. Intracranial electrodes are broadly categorized into subdural electrodes, which are placed on the cortical surface and SEEG, which are placed into deep structures. In recent years, SEEG has been increasingly utilized due to its incrementally decreasing invasiveness and increasing feasibility (Minotti et al., 2018). SEEG along with cortical surface electrodes may allow for high precision and safety in its application (Minotti et al., 2018). Studies have reported that the use of computer-assisted planning during SEEG implantation improves both safety and gray matter sampling (Vakharia et al., 2018). Furthermore, Dasgupta et al. demonstrated that incorporating spatial constraints derived from previous SEEG trajectories enhances the accuracy and safety and speed of computer-assisted planning.

Following intracranial electrode placement, in addition to seizure observation, electrical stimulation can provide critical preoperative information. One key application is functional mapping, which helps delineate motor and language areas before surgery (Baumgartner et al., 2019). In this topic, Lu, Guo et al. analyzed fMRI data from drug-resistant epilepsy patients following SEEG implantation and found that stimulation led to a decrease in functional connectivity strength between the frontal and temporal lobes, regions implicated in seizure onset. Their findings contribute to a deeper understanding of the pathophysiological networks underlying drug-resistant epilepsy.

Surgical treatment is tailored to each patient's seizure type and clinical characteristics. When safe resection is feasible, resective surgery is considered a key intervention for achieving seizure freedom (Ryvlin et al., 2014). However, if the seizure onset zone is not focal or if resection is expected to cause postoperative neurological deficits, alternative treatment options must be explored (Ryvlin et al., 2014). Cottier et al. conducted a systematic review and meta-analysis comparing resective and disconnective surgeries in patients with epileptic spasms. Their findings revealed that while only one-third or less of patients who underwent disconnective surgery achieved seizure freedom, two-thirds or more of those who underwent resective surgery became seizure-free.

Among non-resective treatments, laser interstitial thermotherapy (LITT) has rapidly gained attention as a minimally invasive approach. Compared to resective surgery, LITT has been reported to potentially have a lower risk of cognitive impairment (Drane et al., 2015). Using bibliometric analysis, Chen et al. (2021) visualized the scientific progress and research trends in LITT, highlighting its expanding role in epilepsy treatment, although LITT may not be at present superior to seizure free outcomes from temporal lobectomy and LITT's authoritative outcome and indications are still evolving.

Deep Brain Stimulation (DBS) has also gained increasing recognition as a non-resective approach. In the early 2000s, pilot trials targeting the anterior nucleus of the thalamus (ANT) were conducted in North America (Hodaie et al., 2002; Kerrigan et al., 2004). This led to the publication of the SANTE study in 2010, followed by FDA approval in 2012, after which ANT-DBS became more widely adopted in the United States (Fisher et al., 2010). CM and pulvinar investiagions are currently evolving in treating Epilepsy. However, despite its growing acceptance, global implementation remains limited. Abzalova et al. reported Kazakhstan's first case of ANT-DBS, underscoring its relatively lower adoption outside North America and Europe.

## Conclusion

The studies presented in this Research Topic highlight significant advancements in epilepsy research, encompassing biomarkers, neuroimaging techniques, surgical approaches, and neuromodulation interventions. We hope that readers will find this Research Topic to be a valuable reference for both clinical practice and research.

## References

[B1] Asano E. Juhász C. Shah A. Sood S. Chugani H. T. (2009). Role of subdural electrocorticography in prediction of long-term seizure outcome in epilepsy surgery. Brain 132, 1038–1047. 10.1093/brain/awp025 19286694 PMC2668945

[B2] Baumgartner C. Koren J. P. Britto-Arias M. Zoche L. Pirker S. (2019). Presurgical epilepsy evaluation and epilepsy surgery. F1000Research 8:F1000. 10.12688/f1000research.17714.1 31700611 PMC6820825

[B3] Ben-Menachem E. (2014). Medical management of refractory epilepsy—Practical treatment with novel antiepileptic drugs. Epilepsia. 55, 3–8 10.1111/epi.12494 24400690

[B4] Bettus G. Bartolomei F. Confort-Gouny S. Guedj E. Chauvel P. Cozzone P. J.. (2010). Role of resting state functional connectivity MRI in presurgical investigation of mesial temporal lobe epilepsy. J. Neurol. Neurosurg. Psychiatry 81, 1147–1154. 10.1136/jnnp.2009.191460 20547611

[B5] Binder J. R. Sabsevitz D. S. Swanson S. J. Hammeke T. A. Raghavan M. Mueller W. M. (2008). Use of preoperative functional MRI to predict verbal memory decline after temporal lobe epilepsy surgery. Epilepsia 49, 1377–1394. 10.1111/j.1528-1167.2008.01625.x 18435753 PMC2943848

[B6] Chen Z. Maturana M. I. Burkitt A. N. Cook M. J. Grayden D. B. (2021). High-frequency oscillations in epilepsy: what have we learned and what needs to be addressed. Neurology 96, 439–448. 10.1212/WNL.0000000000011465 33408149

[B7] Crone N. E. Miglioretti D. L. Gordon B. Lesser R. P. (1998). Functional mapping of human sensorimotor cortex with electrocorticographic spectral analysis. II. Event-related synchronization in the gamma band., Brain 121, 2301–2315. 10.1093/brain/121.12.2301 9874481

[B8] Drane D. L. Loring D. W. Voets N. L. Price M. Ojemann J. G. Willie J. T.. (2015). Better object recognition and naming outcome with MRI-guided stereotactic laser amygdalohippocampotomy for temporal lobe epilepsy. Epilepsia 56, 101–113. 10.1111/epi.12860 25489630 PMC4446987

[B9] Falco-Walter J. (2020). Epilepsy—definition, classification, pathophysiology, and epidemiology. Seminars Neurol. 40:1718719. 10.1055/s-0040-1718719 33155183

[B10] Fisher R. Salanova V. Witt T. Worth R. Henry T. Gross R.. (2010). Electrical stimulation of the anterior nucleus of thalamus for treatment of refractory epilepsy. Epilepsia 51, 899–908. 10.1111/j.1528-1167.2010.02536.x 20331461

[B11] Hodaie M. Wennberg R. A. Dostrovsky J. O. Lozano A. M. (2002). Chronic anterior thalamus stimulation for intractable epilepsy. Epilepsia 43, 603–608. 10.1046/j.1528-1157.2002.26001.x 12060019

[B12] Ibrahim G. M. Morgan B. R. Lee W. Smith M. L. Donner E. J. Wang F.. (2014). Impaired development of intrinsic connectivity networks in children with medically intractable localization-related epilepsy. Hum. Brain Mapp. 35, 5686–5700. 10.1002/hbm.22580 24976288 PMC6869397

[B13] Janson M. T. Bainbridge J. L. (2021). Continuing burden of refractory epilepsy. Ann Pharmacother. 55, 406–408. 10.1177/1060028020948056 32795085

[B14] Jiang L. W. Qian R. B. Fu X. M. Zhang D. Peng N. Niu C. S.. (2018). Altered attention networks and DMN in refractory epilepsy: a resting-state functional and causal connectivity study. Epilepsy Behav. 88, 81–86. 10.1016/j.yebeh.2018.06.045 30243110

[B15] Kalamatianos T. Mavrovounis G. Skouras P. Pandis D. Fountas K. Stranjalis G. (2023). Medial pulvinar stimulation in temporal lobe epilepsy: a literature review and a hypothesis based on neuroanatomical findings. Cureus. 15:e35772. 10.7759/cureus.35772 37025746 PMC10071339

[B16] Kay B. P. DiFrancesco M. W. Privitera M. D. Gotman J. Holland S. K. Szaflarski J. P. (2013). Reduced default mode network connectivity in treatment-resistant idiopathic generalized epilepsy. Epilepsia 54, 461–470. 10.1111/epi.12057 23293853 PMC3593969

[B17] Kerrigan J. F. Litt B. Fisher R. S. Cranstoun S. French J. A. Blum D. E.. (2004). Electrical stimulation of the anterior nucleus of the thalamus for the treatment of intractable epilepsy. Epilepsia 45, 346–354. 10.1111/j.0013-9580.2004.01304.x 15030497

[B18] Minotti L. Montavont A. Scholly J. Tyvaert L. Taussig D. (2018). Indications and limits of stereoelectroencephalography (SEEG). Neurophysiologie Clinique 48, 15–24. 10.1016/j.neucli.2017.11.006 29352627

[B19] Morrell M. J. Denison T. (2022). Neuromodulation in 2035: the neurology future forecasting series. Neurology. 98:65 10.1212/WNL.0000000000013061 35263267 PMC8762584

[B20] Motoi H. Miyakoshi M. Abel T. J. Jeong J. W. Nakai Y. Sugiura A.. (2018). Phase-amplitude coupling between interictal high-frequency activity and slow waves in epilepsy surgery. Epilepsia 59, 1954–1965. 10.1111/epi.14544 30146766 PMC6204289

[B21] Nanda P. Sisterson N. Walton A. Chu C. J. Cash S. S. Moura L. M. V. R.. (2024). Centromedian region thalamic responsive neurostimulation mitigates idiopathic generalized and multifocal epilepsy with focal to bilateral tonic-clonic seizures. Epilepsia 65, 2626–2640. 10.1111/epi.18070 39052021

[B22] Ryvlin P. Cross J. H. Rheims S. (2014). Epilepsy surgery in children and adults. Lancet Neurol. 13, 1114–1126. 10.1016/S1474-4422(14)70156-5 25316018

[B23] Sakakura K. Kuroda N. Sonoda M. Mitsuhashi T. Firestone E. Luat A. F.. (2023). Developmental atlas of phase-amplitude coupling between physiologic high-frequency oscillations and slow waves. Nat. Commun. 14:6435. 10.1038/s41467-023-42091-y 37833252 PMC10575956

[B24] Vakharia V. N. Sparks R. Rodionov R. Vos S. B. Dorfer C. Miller J.. (2018). Computer-assisted planning for the insertion of stereoelectroencephalography electrodes for the investigation of drug-resistant focal epilepsy: an external validation study. J. Neurosurg. 130, 601–610. 10.3171/2017.10.JNS171826 29652234 PMC6076995

[B25] Zweiphenning W. Klooster M. A. V. van Klink N. E. C. Leijten F. S. S. Ferrier C. H. Gebbink T.. (2022). Intraoperative electrocorticography using high-frequency oscillations or spikes to tailor epilepsy surgery in the Netherlands (the HFO trial): a randomised, single-blind, adaptive non-inferiority trial. Lancet Neurol. 21, 982–993. 10.1016/S1474-4422(22)00311-8 36270309 PMC9579052

